# Examining the emergence and implementation experience of Primary Health Care Networks (PCNs) in Kenya: a qualitative process evaluation

**DOI:** 10.1186/s12913-025-13652-2

**Published:** 2025-11-18

**Authors:** Beatrice Amboko, Jacinta Nzinga, Anita Musiega, Benjamin Tsofa, Peter Mugo, Rahab Mbau, Anne Musuva, Felix Murira, Ethan Wong, Caitlin Mazzilli, Wangari Ng’ang’a, Nirmala Ravishankar, Salim Hussein, Edwine Barasa

**Affiliations:** 1 Health Economics Research Unit, KEMRI-Wellcome Trust Research Programme, Nairobi, Kenya; 2 Liverpool School of Tropical Medicine, Liverpool, UK; 3 Bill and Melinda Gates Foundation, Seattle, USA; 4 Think Well Global, Nairobi, Kenya; 5 Division of Primary Health Care, Ministry of Health, Nairobi, Kenya; 6 Nuffield Department of Medicine, University of Oxford, Oxford, UK

**Keywords:** Primary Health Care Networks (PCNs), Networks of care, Primary Health Care (PHC), Process evaluation, Implementation experience, Kenya

## Abstract

**Background:**

Kenya has identified Primary Health Care Networks (PCNs) as a key reform to strengthen Primary Health Care (PHC) delivery and enacted the Primary Health Care Act of 2023 to support their implementation. PCNs were piloted in Kisumu and Garissa counties in 2020 and rolled out nationally in 2023. However, little is known about how PCNs are being implemented across diverse county contexts. This study examined the emergence and implementation experience of the PCN reform in Kenya.

**Methods:**

We used a cross-sectional qualitative process evaluation design. We collected data at the national level and in five purposefully selected counties, using in-depth interviews (*n* = 65) and document reviews, between February and June 2024. Participants included stakeholders from the national level (Ministry of Health, development and implementing partners, and the Council of Governors), county level (county health departments, sub-county managers, multi-disciplinary team (MDT) members, facility managers, and frontline health workers), and community level (community health committee chairs and community health workers). We reviewed policy documents and county reports on PCN implementation for document review. We analysed the data using a thematic approach.

**Results:**

The emergence of PCNs as a policy reform was motivated by a technocratic process that identified underlying challenges in PHC service delivery and proposed PCNs as a solution, as well as political interest and support that facilitated their adoption. The implementation effectiveness of PCNs varied across the study counties, with critical aspects of PCN design, such as the establishment of MDTs and the digitisation of PCNs, being inadequately implemented. The effectiveness of PCNs’ implementation may have been constrained by capacity gaps in key foundational aspects of PHC health systems, including financing, human resources, health commodities, and information systems. Moreover, the implementation effectiveness of PCNs may have been undermined by the limited integration of key health facility functions, including financing, human resource management, health commodity supply chains, information systems, and care coordination.

**Conclusion:**

Strengthening PCN implementation in Kenya requires investment in policy capacity to ensure effective implementation. The foundational aspects of PHC systems must be reinforced. The PCN design should be refined to enhance the integration and coordination of key health facility functions.

**Supplementary Information:**

The online version contains supplementary material available at 10.1186/s12913-025-13652-2.

## Background

Primary health care (PHC) is widely recognised as a critical pathway to achieving universal health coverage (UHC) as it offers a cost-effective and equitable approach to delivering essential health services [1]. Kenya has prioritised strengthening its PHC system, aiming to provide comprehensive, patient-centred, and integrated services. Ensuring high-quality care requires systematic coordination across various service delivery points. These include the integration of community-level interventions, which are increasingly acknowledged as essential components of effective health systems [2].

Kenya has a devolved governance structure consisting of a national government and 47 semi-autonomous county governments [3]. The counties primarily manage health service delivery. Counties oversee primary and secondary care, manage health facilities, and recruit healthcare workers [4]. The national government is responsible for policy, regulation and tertiary care. Both public and private sectors contribute to service delivery, with the private sector owning 54% of all health facilities as of 2023 [5]. Health services are structured into four tiers and six levels. Tier 1 includes community health services (CHS), delivered through community health units (CHUs) at the level 1. Tier 2 consists of primary care facilities, such as dispensaries (Level 2) and health centres (Level 3). Tiers 3 and 4 include county referral hospitals (levels 4 and 5) and national referral hospitals (level 6), respectively [6]. Despite this structure, fragmentation, weak referral linkages, and resource imbalances have constrained the delivery of high-quality PHC [2].

Kenya’s PHC reorganisation has been closely tied to its broader UHC agenda, particularly after UHC was politically prioritised under the Big Four Agenda in 2018 [2, 7]. This was followed by the launch of the UHC pilot in four counties, which highlighted systemic challenges such as poor trust in PHC facilities, weak referral mechanisms, and poor linkage with communities, prompting a shift toward more integrated service models [8, 9]. This led to the development of Primary Health care Networks (PCNs) in 2020 as a key reform for delivering PHC [2, 8]. PCNs were designed to link CHUs with primary care facilities to improve access, strengthen coordination, and ensure community-responsive services. As a result, PCNs have become a central reform embedded within Kenya’s UHC strategy to promote service integration, efficiency, and financial protection [2, 8, 10]. The PCN model is designed to enhance service delivery by strengthening referral systems, improving the efficiency of the health system, and ensuring equitable access to quality healthcare. Importantly, PCNs aim to integrate public, private, and CHS under a unified network and promote community participation in health planning and service delivery [2, 8].

### Primary Health Care Networks (PCNs) in Kenya

**Fig. 1:**
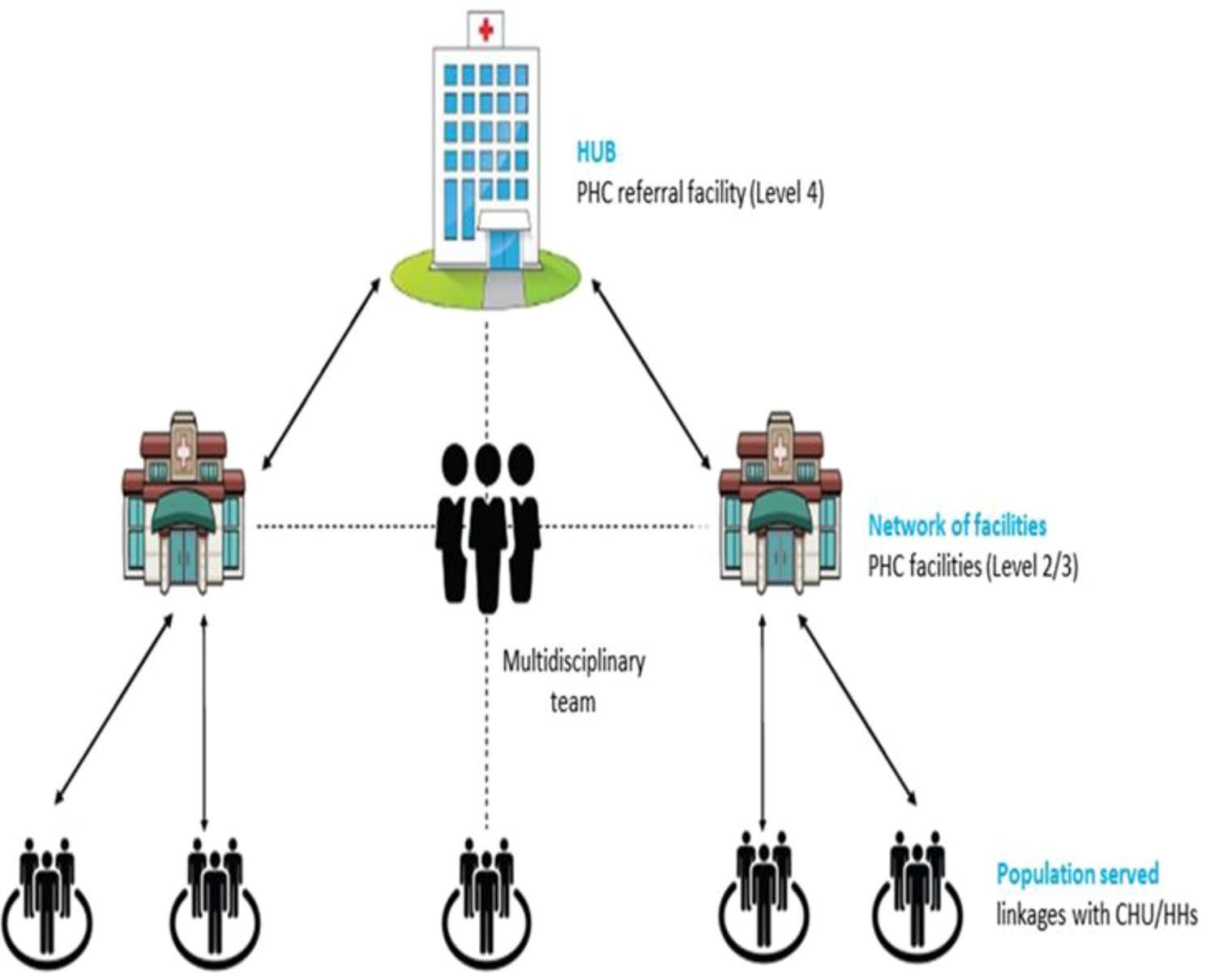
Proposed model of the Primary Health Care Network (PCN) – ‘Hub and spoke model’ [8]

In 2020, Kenya’s Ministry of Health (MoH) proposed the creation of PCNs at the sub-county level. PCNs are geographically defined groups of healthcare facilities and CHUs, linked through a common administrative and clinical governance framework to deliver coordinated, comprehensive PHC services [11–13]. These networks consist of a PHC referral facility (level 4 hospital (public, private or faith-based)), serving as the hub, and several other PHC facilities (levels 2 and 3 (public, private or faith-based)) functioning as spokes and CHUs, forming an integrated PHC system (Fig. 1) [2, 8]. The hubs function as the initial referral level within counties and are responsible for supporting spokes. This structure ensures that patients receive timely and appropriate care at all levels of the healthcare system [8]. In 2023, the Kenyan government enacted the Primary Health Care Act 2023, aimed at establishing a regulatory framework for the implementation and nationwide expansion of PCNs [14].

A PCN should be managed by a multi-disciplinary team (MDT) with diverse expertise and skills, led by a family physician. A functional PCN requires a PCN coordinator, a structured referral system, well-defined facility and community linkages, and integrated facility functions. To ensure the continuous delivery of essential PHC services, PCNs should optimise available regional resources, including human resources, infrastructure, health products, technology, finance, and governance, to effectively meet community healthcare needs [8]. The national target is to establish at least one PCN per subcounty, totalling 315 PCNs, and as of February 2025, 221 PCNs had been established with support from various partners. This rapid rollout followed the 2023 Primary Health Care Act, which positioned PCNs as the foundation for integrated PHC in Kenya. However, there is limited evidence on how these networks are being implemented across counties under a devolved governance system. This study aimed to examine the emergence and early implementation experience of PCNs in Kenya, providing critical evidence to refine the design and execution of PCN reforms while offering insights for similar health system reforms in comparable settings.

## Methods

### Conceptual framework

**Fig. 2:**
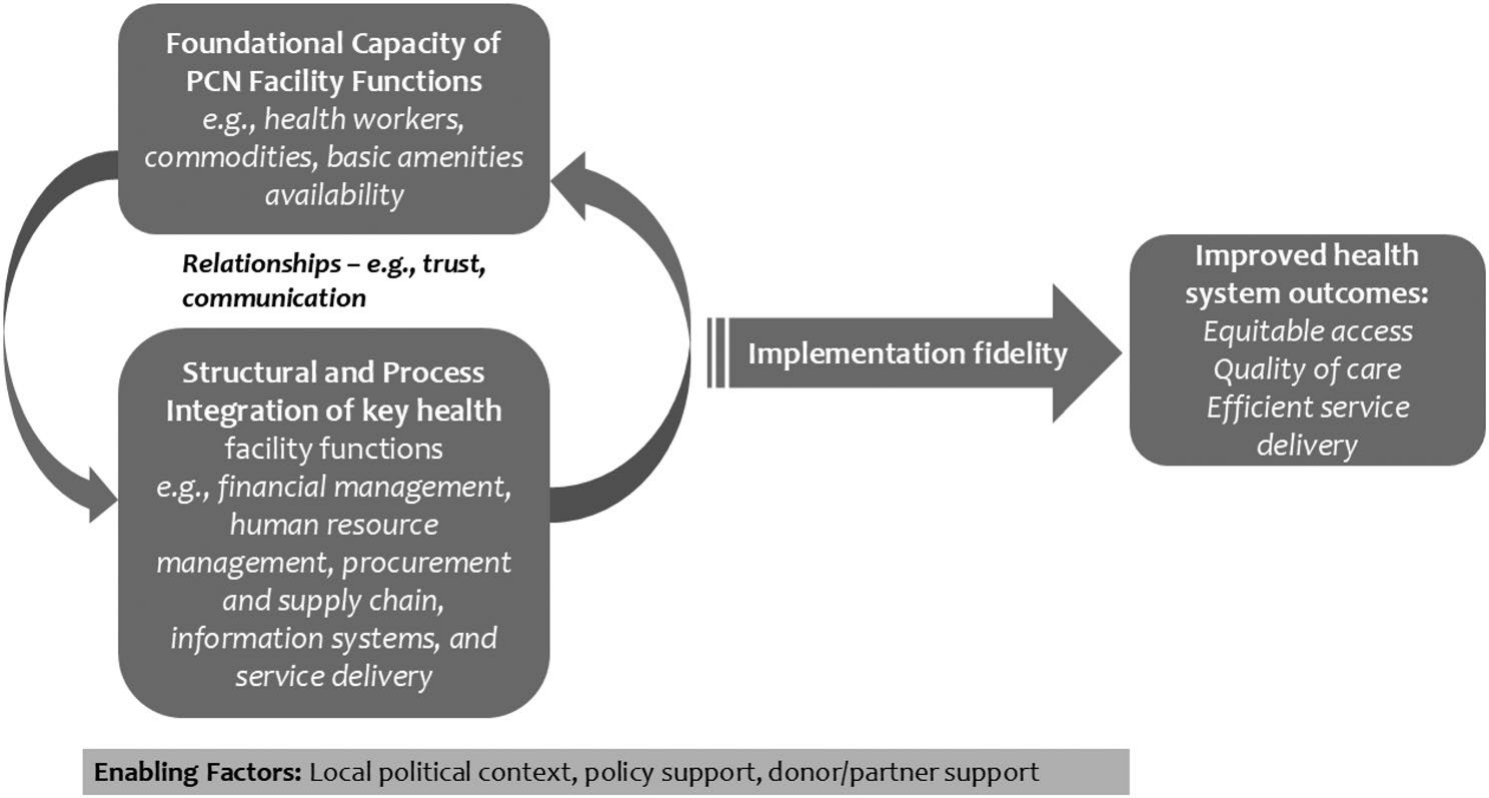
Conceptual framework

This study was guided by a conceptual framework (Fig. 2) that made the following assumptions. First, for PCNs to function effectively, the foundational capacity of individual health facilities within the network must be optimal. Specifically, critical facility functions, including financing, human resources, health infrastructure, commodities and supply chain management, information systems, and service delivery, must be operating at an optimal level or be strengthened before the introduction of PCNs. This foundational capacity is essential for ensuring that the intended outcomes of PCNs, such as efficiency, equity, quality, and access, are effectively realised.

Second, our framework assumes that the operationalisation of PCNs as a coordinated network of care requires structural and process-relatedness, as well as coordination among the health facility functions. For instance, financial management, human resource management, commodities and supply chain management, information systems, and service delivery should be integrated. Effective coordination may involve establishing a financial relationship that integrates revenue generation, resource allocation, and expenditure. For example, a PCN could be designed as a budgeting and expenditure unit, where facilities jointly budget and allocate resources. Similarly, coordinated human resource and health commodities management should enable the sharing and allocation of staff and resources across the network rather than confining them to individual facilities. Ideally, a PCN would also employ an integrated information system that ensures seamless information flow and visibility across all participating facilities.

Third, while this structural and process-relatedness and coordination addresses the hardware components of the PCNs, the software elements, specifically the relationships among actors within the PCNs, are equally critical. For instance, to foster effective collaboration and cohesiveness in implementing the structural and process elements, trust among staff and managers across the various PCN health facilities is necessary. Fourth, to effectively operationalise both the foundational capacity of PCN facilities and the integration of structural and process elements, it is essential to ensure implementation fidelity or operational effectiveness. Lastly, all these assumptions can only be achieved in an enabling environment that influences foundational capacity, integration, and relationships. Contextual elements, such as local political dynamics, governance, and donor/partner support, shape the conditions under which PCN functions are implemented and sustained (Fig. 2).

### Study design

This study used a cross-sectional qualitative process evaluation to assess the emergence and implementation experiences of PCNs. Comprehensive details on the methodology are outlined in the published study protocol [15]. In summary, the evaluation was guided by a conceptual framework developed for this study (Fig. 2), which focused on three key components: [1] foundational capacity of individual health facilities (including infrastructure, staffing, and commodity availability); [2] functional integration of facility services across PCNs (such as referral systems, data systems, and human resource sharing); and [3] fidelity of implementation, assessing adherence to national PCN guidelines, reach, and unintended consequences. These components were analysed within the broader policy and implementation context. The evaluation also explored how PCN reforms emerged, what drove their adoption, and which factors enabled or constrained implementation [16]. Data collection involved a combination of document reviews and in-depth interviews with key informants to provide a comprehensive understanding of the PCN implementation process.

### Study sites

We collected data from 5 purposively selected counties in collaboration with the MoH. The selection of counties considered geographical diversity, level of PCN implementation, and the diversity of funders and partners supporting the establishment of PCNs. To ensure participant confidentiality, we have anonymised the counties involved in the study. Table 1 outlines the characteristics of the selected counties and provides justification for their selection.

**Table 1:** Characteristics of study counties

County	Population size (2019 census)	Justification of selection	Funder and partners supporting PCNs	Year started PCN implementation	PCN implementation status
County A	1,155,574	It is a peri-urban county from the Western region. It was one of the counties where PCNs were piloted in 2020, and it has established all PCNs across all seven sub-counties, supported by UNICEF and other partners. Additionally, the county demonstrates a strong political commitment to PHC.	UNICEF	2021	100% (7/7)
County B	841,353	It is a marginalised, predominantly rural, semi-arid county in the Northeastern region. It was one of the counties where PCNs were piloted in 2020, and it is the only county where all PCNs were established and gazetted across all the seven sub-counties supported by UNICEF.	UNICEF	2020	100% (7/7)
County C	866,820	It is a rural and urban county in the Coastal region. Four out of five PCNs have been established and supported by Amref and the county.	Amref	2022	80% (4/5)
County D	2,162,202	It is a peri-urban county located in the Rift Valley region. It had one smart PCN established in one sub-county supported by USAID and ThinkWell.	USAID Tujenge Jamii	2023	100% (11/11)
County E	987,653	It is a rural county in the Eastern region. It has established all PCNs across all six sub-counties led by family physicians, supported by PATH, CIHEB and ThinkWell.	PATH	2023	100% (6/6)

### Study participants

We collected data from 65 purposefully selected participants from national, county, facility, and community levels involved in PHC decision-making, implementation, and delivery. Table 2 outlines the characteristics of the study participants.

**Table 2:** Summary of study participants

Level of respondents	Male	Female	Total
**National level**			
Partners	6	4	10
Council of governors	1	0	1
**County level**			
County Department of Health	14	3	17
Sub County Managers	7	0	7
MDT members	1	2	3
Health Facility Managers	7	5	12
Frontline health workers	1	6	7
Community Health Committee (CHC) Chair	3	0	3
CHWs	4	1	5
**Total**	**44**	**21**	**65**

### Data collection

We collected data between February and June 2024 through document reviews and in-depth interviews (IDIs). The IDIs were conducted in English and Kiswahili using a semi-structured interview guide (Supplementary file 1). All study participants were provided with an information sheet, and consent was sought for participation in the interviews. The interviews were conducted face-to-face at the participants’ workplaces and audio-recorded using encrypted audio recorders. We employed data saturation to guide our qualitative sample size. Saturation was assessed iteratively during data collection and analysis and was considered achieved when no new themes or information emerged from the interviews. The documents reviewed comprised the national PHC strategic framework, PCN guidelines, PHC Act 2023, biweekly PCN implementation meeting minutes, and county PCN reports.

### Data management and analysis

Audio recordings from the IDIs were transcribed verbatim in their original language and translated into English as needed, with each transcript reviewed against its corresponding audio file to ensure transcription accuracy. Validated transcripts were then imported into NVivo (version 12) for coding and thematic analysis. The coding framework was based on (a) a priori issues derived from the original research objectives and incorporated into the interviews, (b) emergent issues identified by the respondents, and (c) analytical themes that emerged from the repeated expression of specific views or experiences deemed significant and relevant.

The desk review involved systematic content analysis of key policy and program documents, including the national PHC strategic framework, PCN guidelines, biweekly PCN implementation meeting minutes, and county PCN reports. Information was extracted using an Excel matrix organised around our study objectives and pre-identified thematic areas. This facilitated structured data capture and analysis, enabling us to identify key themes and align document insights with findings from the in-depth interviews for triangulation and validation.

### Ethical considerations

This study received ethical approval from the KEMRI Scientific and Ethics Review Unit (SERU) under approval number KEMRI/SERU/CGMR-C/294/4708, as well as clearance from the Council of Governors, Kenya, and the National Commission for Science, Technology, and Innovation (NACOSTI), reference no. 296,109.

## Results

This section presents findings from the process evaluation, organised thematically around the emergence of the PCN reform, fidelity of implementation, foundational service delivery capacity, functional integration, implementation context, and key facilitators. These themes were developed through iterative thematic analysis of interview transcripts and document reviews.

### Emergence of the PCN reform

**The PCN reform in Kenya had both technical and political motivations.** The technical motivation stemmed from the challenges identified in previous attempts to implement UHC reforms in Kenya. These included a tendency for patients to bypass PHC facilities to seek PHC services at higher-level facilities due to a lack of trust in PHC facilities and inadequate service provision at the PHC level. Moreover, there was weak integration between the community level and PHC facilities [8]. In response to these gaps, the Kenya MoH initiated PCN pilots in two high-burden maternal mortality counties, Kisumu and Garissa, in early 2020. These efforts were formalised with the development of PCN operational guidelines in 2021.

*When the UHC pilot was done*,* everyone went to level 5 facilities*,* bypassing lower-level health facilities. The whole point of PCNs is to ensure that facilities in the entire network are supported to be able to provide basic healthcare services so that*,* for example*,* you don’t have to go to a level 5 healthcare facility when you have a simple ailment such as a flu*. **IDI2_Female Maternal and Newborn Health (MNH) Lead Partner B**

*Two counties were identified for the PCN pilot; that is where the PCN journey began. PCNs were intended to improve service delivery at the primary health level and ensure that the residents of the county can attain the highest possible form of quality care even at lower-level health facilities.* **IDI1_Male PHC Coordinator County A**

The PCN reform was also motivated by political interest. The current ruling party, *Kenya Kwanza*, included PHC reforms as part of their political manifesto. With the coming to power of the Kenya Kwanza government in August 2022, PCNs were identified as a key strategy for implementing PHC. This was solidified with the enactment of the PHC Act in 2023, which mandated all counties in Kenya to implement PCNs as a model for delivering PHC services nationwide [14].

*…the bills that were signed into law by His Excellency*,* the President of the country*,* William Samoei Ruto*,* just before Mashujaa Day last year. So*,* we have the primary health care bill*,* we have the restructuring of the National Health Insurance Fund (NHIF) basically to the Social Health Insurance Fund (SHIF)*,* as managed by the Social Health Authority (SHA)*,* and then we have the digital bill and*,* you know*,* that…that kind of restructuring*,* is what is deliberately meant to ensure that we focus more on the primary health care*,* the preventive medicine*,* as opposed to the curative medicine*,* as has been the case*,* of course not forgetting the curative medicine*,* we can’t forget treatment.* **IDI7_Male County Executive Committee member for Health County D**

### Fidelity of implementation

#### PCN legislative and policy frameworks

**Legislative and policy frameworks underpinned the PCN reform.** The Kenya Primary Health Care Strategic Framework 2019–2024 identified PCNs as a key strategy to advance PHC goals in Kenya [2]. The Kenya Community Health Policy 2020–2030 and the Kenya Community Health Strategy 2020–2025 provided the policy direction and strategies to strengthen CHS and their linkage to facility-based PHC service delivery, which form a key component of PCNs [17, 18]. Complementing these overarching PHC and CHS policies and strategies are specific PCN guidelines and the PHC Act 2023, which provide legal and operational directives for establishing and governing PCNs [8, 14]. These documents underscore a cohesive approach to integrating and regulating PCNs within Kenya’s health care delivery system.

*Of course*,* the implementation is guided by the National strategic plans. Initially the national strategic plans*,* now we have the PHC Act of 2023.* **IDI1_Female PHC Coordinator County B**

#### PCN establishment process

Table 3 outlines the steps that counties need to undertake to establish PCNs and the status of implementation across the study counties.

**Table 3:**
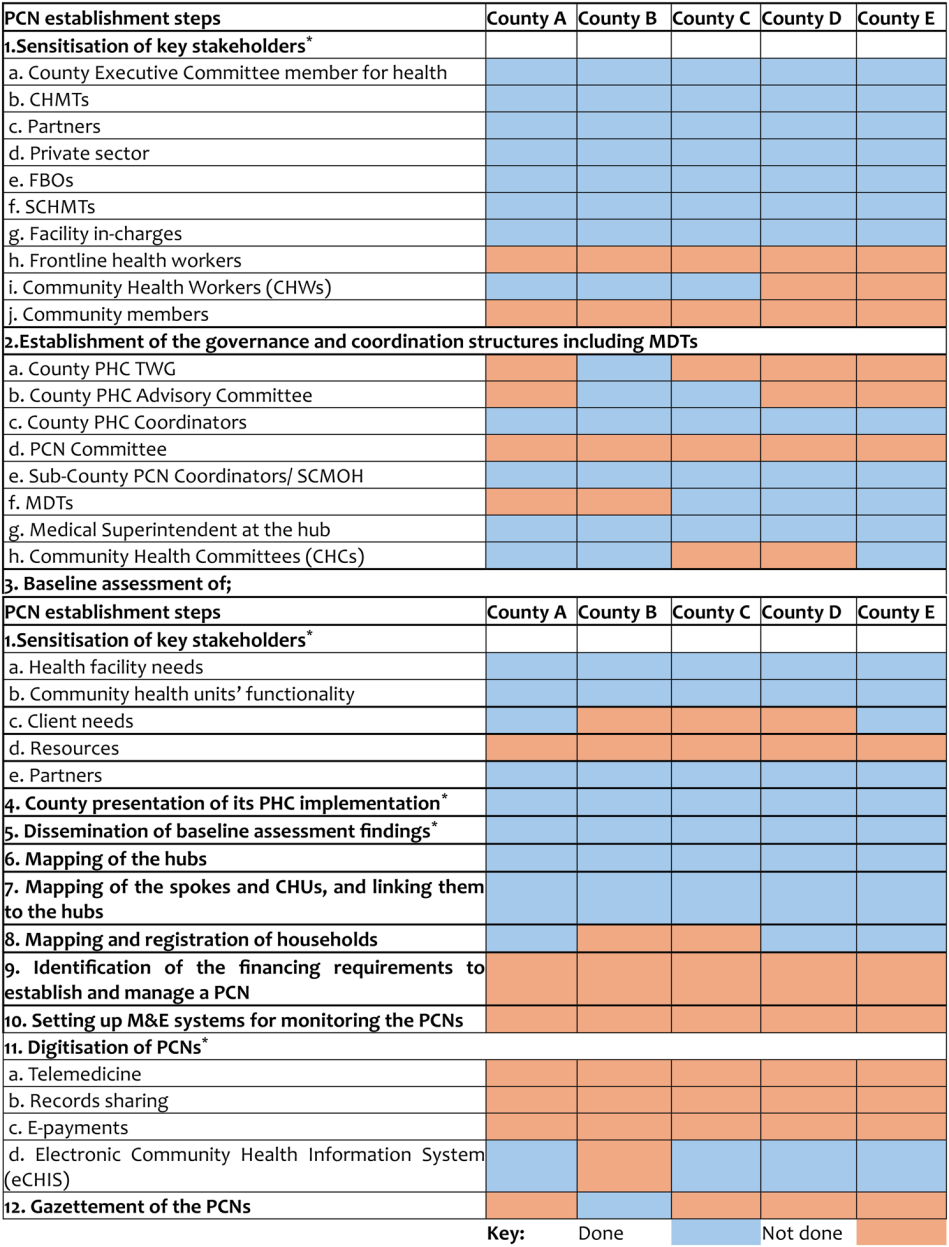
PCN establishment steps across the study counties as of June 2024 [8]

^*^ The additional steps were obtained from presentations at various meetings by the national MoH and reflects the actual steps followed by counties to establish PCNs

**Sensitisation of the reform targeted multiple levels of the health system at the county level**. All study counties began implementation by sensitising targeted stakeholders on the PCN reform. The sensitisation was undertaken by the national MoH, partners, county and sub-county trainers of trainers for over one to two days. Sensitisation was more extensive at the higher levels of the county health system. **There was inadequate sensitisation of frontline health workers**,** community health workers**,** and community members.** This was reported to be due to resource constraints.

*…we started sensitising the executive*,* and then we went to the county health management team (CHMT)*,* then we went to the sub-county Health Management Team (SCHMT)*,* … after the sub-county HMT we went to the MDT leads*,* then the MDTs then the facilities…* **IDI6_Male PHC Coordinator County D**

*There was just a small sensitisation*,* a one-day sensitisation that involved a team from the MOH and the county team led by the PCN focal person*,* Dr. XXX*,* who did some little bit of training. But we feel that this team [SCHMT] needs to be capacity-built more so that they can understand their roles.* **IDI10_Male SCMOH County D**

**Fig. 3:**
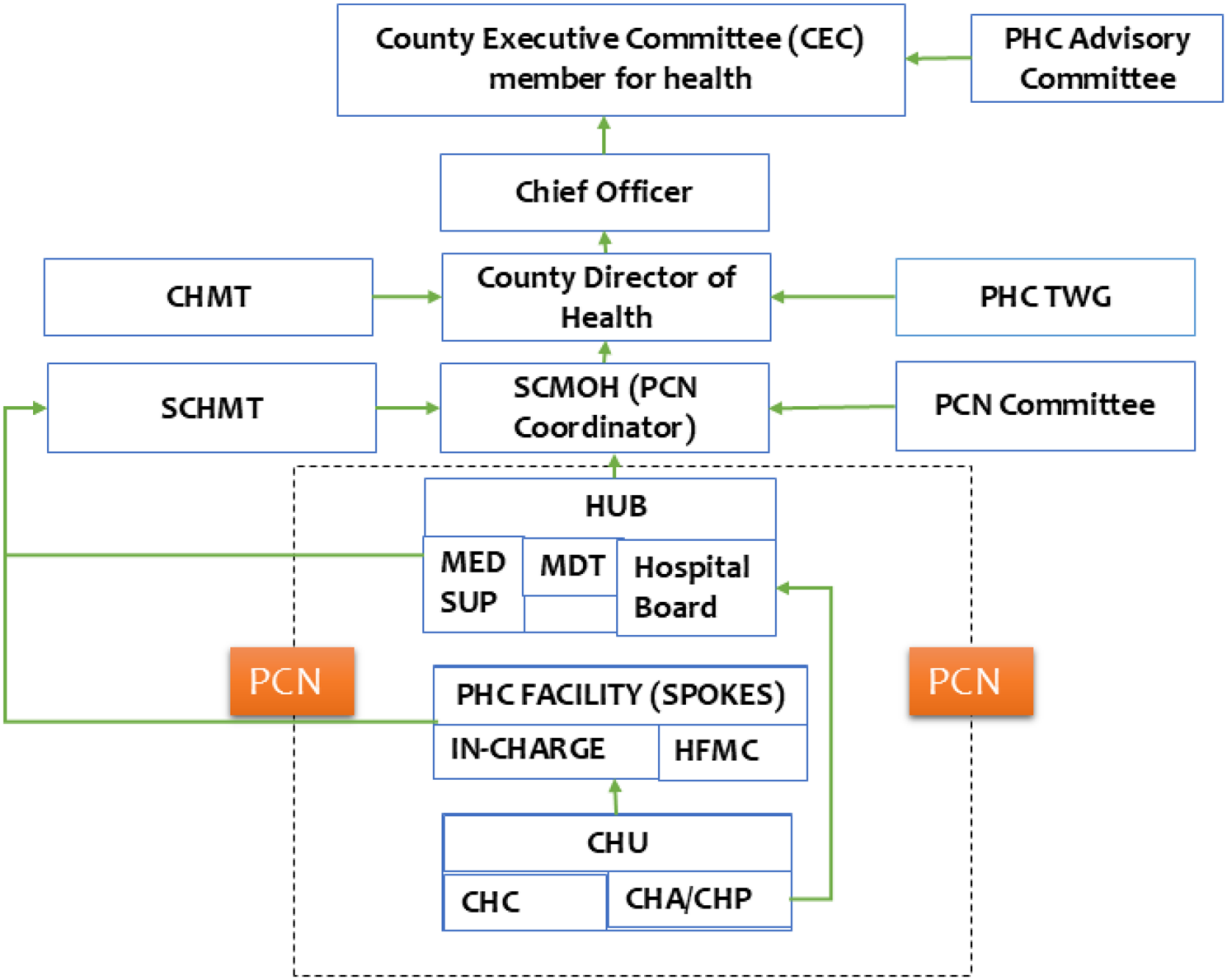
PCN governance and accountability structures [8]

**There were gaps in the establishment of PCN governance structures across the study counties**. Figure 3 outlines the governance structure of a PCN as per the guidelines. All counties had County and Sub-County PHC coordinators and Medical Superintendents (Medsups) at the Hub facilities. These structures predated the establishment of PCNs. The establishment of all other required governance structures varied across counties. For instance, the establishment of multi-disciplinary teams (MDTs) to coordinate team-based care in the PCNs was only achieved in three out of the five study counties. In comparison, the establishment of County PHC technical working groups (TWGs) and County PHC advisory committees were only achieved in one out of the five study counties. This incompleteness in the establishment of PCN governance structures was attributed to a lack of political support and resources to facilitate their operationalisation.

*…the establishment of governance and coordination structures is something that is supposed to come preliminary in the early stages of implementation. But then*,* because of politics and issues in the county government*,* sometimes you want the County Executive Committee (CEC) members for health to talk to other CECs and help you put up an advisory council. And they are all engaged*,* and you then decide to continue with other steps as those others are being put in place.* **IDI10_Male Senior Technical Advisor Partner J**

**All study counties conducted baseline assessments before the establishment of PCNs.** This included an assessment of development partners to support PCN implementation and the needs of healthcare facilities and CHUs. However, only two counties assessed client needs, and none assessed the resources needed to establish and implement PCNs. Counties reported using these baseline assessments to inform the mapping of facilities within PCNs.

*We did a baseline survey where we went to all the six sub-counties and select facilities … we had several tools we were using … a client exits tool*,* facility assessment tool and the community assessment tool …* **IDI6_Male PHC Coordinator County D**

**All study counties successfully mapped and established connections between their hubs**,** spokes**,** and CHUs**. Adherence to the hub-and-spoke model was widespread among the counties, although two counties adapted this model by incorporating mini-hubs. A mini hub is either a high-volume level 4 or level 3 facility. This adaptation was necessitated by political interests that required an equitable establishment of hubs across different geographical regions within the counties. Other reasons included the need to address the long distances to hub facilities and the varying demographic characteristics, such as refugee camps and nomadic populations. The linkages spanned government, faith-based organisations (FBOs), and non-governmental organisations (NGOs) facilities.

*…things are not cast in stone*,* and each county and each population have its own different dynamics. It’s not one-size-fits-all; for example*,* the idea of sub-hubs is not there at the national level. The only thing that the national recommends is you need to have a hub and spoke model*,* but we’ve introduced another layer of the sub-hub*,* and so there are things we are looking at; number one is involving the key players*,* the political class in the health agenda.* **IDI1_Male PHC Coordinator County A**

**None of the study counties reported identifying the financial requirements for establishing and managing PCNs**. They all relied on estimates from the national MoH where four scenarios were considered: (1) the total cost assuming zero investments (USD 10,410,518), (2) the cost based on needs and gaps using incremental costing (USD 1,526,439), (3) the cost assuming the current human resources for health (HRH) and infrastructure are adequate (USD 759,656), and (4) the cost assuming HRH, commodities/supplies, and infrastructure are available (USD 202,256). Moreover, none of the study counties had developed a sustainable financial plan for the operationalisation of PCNs. There was also a lack of established **monitoring and evaluation systems** for PCNs across all the study counties due to a lack of guidance from the national MoH. This indicates a gap in both financial planning and oversight mechanisms, which are critical for the effective implementation and management of PCNs.

**Finally**,** the digitisation of PCNs was found to be inadequate**. Four of the study counties had implemented electronic Community-Based Health Information Systems (e-CHIS) to digitise their community health systems. Additionally, **only one county had officially gazetted** its PCNs, having successfully established all PCNs within its jurisdiction (Table 3).

### PCN foundational capacity and integration

#### Governance and accountability

**The planning and performance management of health facilities was not integrated as a PCN.** The PCNs were expected to undertake joint annual work planning (AWP), budgeting and priority setting to enhance their efficiency [8]. However, none of the study counties had done this. Further, health facilities within PCNs did not develop joint performance targets. Each facility in the PCNs maintained its previous annual work planning and performance target development process, where it developed its own. None of the study counties reported undertaking performance management of PCNs.

*We have the community health units; they have their own annual work plan*,* which is consolidated. We have the spokes; they have their own annual work plans. And then we have the sub-county annual work plan.* **IDI1_Male Sub County Team Lead County E**

**Multi-disciplinary teams (MDTs) did not play their supportive supervision roles for PCN health facilities**. MDTs were expected to provide supportive supervision to the spokes health facilities in PCNs. There was a lack of clarity regarding the role of the MDT supportive supervision role and that of sub-county health management teams (SCHMTs), which were traditionally tasked with conducting quarterly supportive supervision visits to individual health facilities. Moreover, the MDTs did not recognise supportive supervision as part of their role, probably because it is the role of SCHMTs.

*Our roles are just … taking the services to the periphery. Mostly*,* when we go to the periphery*,* we go for the chronic cases; it’s not just a mere outreach. We are supposed to see all outpatient cases. No*,* we only go there when it’s a dispensary where it’s only manned by a nurse; there are those conditions that he or she might not be able to handle. So*,* in such cases*,* she’s supposed to book them*,* then liaise with us and go and review them at the facility.* **IDI8_Female MDT Member County C**

#### Financing

**PCN implementation largely relied on funding from non-state actors.** International and local non-state actors (development and implementing partners) provided both technical and financial assistance to counties to set up and operationalise PCNs.

*We have played a role of…supporting capacity building across the various levels from the county to the community. We have also played a role of … helping them to engage… the legislators*,* to sensitise them on PCN and formation of multi-disciplinary teams…* **IDI15_Female Senior Technical Lead Partner A**

*…we have partners who have stepped in to take care of … some of the gaps. When you go for those outreaches … some partners will pay the staff allowances. I think XXX Kenya is doing that. I’m also aware that through the county government*,* we also have other partners who are coming in*,* and the aim is to bridge the gaps where they have been noted.* **IDI3_Male Medical Superintendent County D**

**The study found that county governments did not have dedicated budgets for PCN setup and operations.** According to PCN guidelines, counties were expected to allocate funds in their budgets for PCN activities. However, four of the study counties did not have an explicit budget designated for PCNs. Only one county had established a budget line for PHC that included a PCN sub-program due to the high political prioritisation of PHC. The absence of a dedicated funding allocation contributed to delays in PCN implementation and limited the scope of PCN activities.

*No*,* we don’t have [a PCN budget]. In fact*,* that is the biggest issue because we want to do an activity*,* but we don’t have a budget. Like these outreaches*,* it is now the sub-county. I’ve gone through the sub-county to see if we can get the drugs and all the HPTs that we need. But here*,* we don’t have any budget for that.* **IDI5_Female MDT Team Lead County E**

**The budgeting process for PCNs was not integrated.** The existing budgeting framework, guided by the Public Finance Management (PFM) Act, did not recognise PCNs as a distinct budgeting entity. Instead, PCN facilities prepared separate budgets, limiting the integration and coordination of resource allocation and prioritisation within PCNs.

*You can only consolidate in the primary healthcare budget when you have one source of financing*,* right? But when you have each facility receiving money on their own*,* they’ll come up with their needs.* **IDI1_Male Sub-County Medical Officer of Health (SCMOH) County B**

*I don’t think that the facilities*,* the spokes*,* had a specific budget for PCN. They just had their normal budget for running the facility. That’s why the Subcounty team has now taken on that role to make the AWP PCN part of it. We have not taken the burden to the spokes or the main hub.* **IDI6_Male SCMOH County C**

**Outreach activities led to revenue losses for hub facilities**. Hub facilities charge for services offered to clients, while services provided by spoke facilities and in the community are expected to be provided at no cost. An unintended outcome of the PCNs was the loss of revenue by hub facilities when they provided health services in spokes or in the community as part of outreach activities, as they were then required to offer these services for free.

*Currently*,* the hubs*,* which are the hospitals*,* charge*,* for example*,* for diabetes and hypertension drugs. The same if availed in the PCNs or in the dispensary*,* they are not charged. So*,* whenever patients hear there is an outreach*,* they rush there to get the free drugs. Okay. So*,* the hospital is losing revenue while clients are getting assisted.* **IDI1_Male County Director of Health (CDH) County D**

#### Health workforce

**Across all the study counties**,** the implementation of PCNs was handicapped by a shortage of healthcare staff**,** particularly in rural and under-resourced areas**. The effective implementation of PHC through PCNs required strengthening the staffing of PHC facilities to meet the capacity requirements for the anticipated workload. Of the study counties, four reported no changes in the number or distribution of health workers. Many facilities, especially those at lower levels of the healthcare system, are operated by just one or two staff members.

*…the challenge we have with some of the facilities is the staff shortage*,* and I think this is not unique because you might find a dispensary which has one nurse*,* and she’s the only technical person in that facility. So*,* if she’s out of the station for a particular reason*,* maybe on leave or on assignment*,* the facility closes.* **IDI7_Male County Executive Committee member (CECm) for Health County D**

Only one study county reported that consultants, such as surgeons and obstetricians, were posted to sub-county hubs for the first time, allowing for general surgeries and caesarean sections to be performed locally.

*Before the PCN*,* there was no surgeon*,* there was no paediatrician*,* there was no gynaecologist; currently*,* in the establishment of the PCN*,* we got the specialists*,* and we still expect more…* **IDI10_Male SCMOH County B**

**Health workers in MDTs experienced an increased workload because of expanded responsibilities.** This was because MDT members were expected to provide services not only at the hubs but also during outreach activities to spoke facilities and in the community. These additional responsibilities, combined with their regular duties, exacerbated the strain on healthcare staff.

*…you believe that when the staff is one*,* and the workload is high*,* the quality will also be compromised*,* so we must also increase the human resource aspect if the workload will be increasing.* **IDI10_Male SCMOH County B**

*The other challenge is staffing*,* where for us to send a team*,* we have to leave other people offering services*,* and most of the level four people are strained in terms of staffing. So that is why we are not even able to do this more frequently*,* as these staff are also expected to offer services within the hospital.* **IDI3_Male Medsup County D**

**The study counties reported a lack of adequate support for MDTs**. Designed to support lower-level facilities by providing integrated care and reducing unnecessary referrals to higher-level hospitals, MDTs were hindered by financial constraints and inadequate logistical support. Without these resources, MDTs were unable to operate effectively.

*…one of the issues that we’ve had is that there is no dedicated financing to PCNs. That’s the biggest challenge. So*,* the movement for these specialised groups from the hubs to the spokes has been restrained because of budget and resource needs.* **IDI1_Female PHC Coordinator County B**

*Another challenge is that we normally go late for the PCN activities because of transport challenges*,* because we sometimes are forced to go with the ambulance*,* and you know the work of an ambulance. So*,* in case of an emergency*,* you’ll have to wait; first*,* you take the emergency to XXX*,* around 11*,*12*,* is when it comes back to take us.* **IDI8_Female MDT member County C**

**PCNs did not have an effective mechanism for sharing health workers**. According to PCN guidelines, team-based care across PCN facilities was intended to be operationalised through MDTs. However, across the study counties, there was significant variation in the understanding of MDTs, and where MDTs existed, they were not fully operational because of resource shortages. Only one county organised consultants to visit hubs, and general practitioners handled issues at lower-level facilities. Half of the counties, especially the early adopters, did not have active PCN MDTs, often citing operational challenges that left these teams inactive, forming them only when necessary. Sharing HRH between hubs and spokes was achieved mainly through referrals and outreach programs.

*The MDTs are not permanent. We form them based on the needs that arise within the community and the sub-county. So*,* it is the hub that most of the time comes up with these ideas.* **IDI8_Male Medsup County A**

*… specialised doctors and consultants are going to our sub-county health facilities weekly. We have a gynaecologist going to that facility on Monday*,* for example*,* we have a surgeon going on Tuesday…prior to that*,* the medical officer who is there tries to register all those patients*,* bring them together*,* prepare them for the respective specialisation and all the consultants.* **IDI8_Male SCMOH County B**

**MDT outreach activities disrupted service provision at hub facilities.** All the study counties with active PCN MDTs reported disruptions to service provision at hub facilities. This occurred because MDT members, whose roles originally entailed providing services at the hub facilities, were now required to provide outreach services to spoke health facilities without the recruitment of additional staff to cover the staff engaged in outreach services.

*…because the [staff that are] part of the MDT are also our staff at the facility. So*,* you find a time when that staff member is on duty here*,* and the PCN also wants him to join. So*,* sometimes you get that challenge that this person also has to be offering services around here*,* and he has to go to PCN.* **IDI6_Male SCMOH County C**

#### Procurement and supply chain for health commodities

**Inadequate availability of health commodities compromised PCN service delivery across all the study counties**. The delivery of PHC services, especially in lower-level health facilities (health centres and dispensaries) within the PCNs, was compromised by chronic shortages of essential medicines, diagnostics, and medical equipment. This limited the scope of services that lower-level PHC facilities could provide, forcing patients to seek referrals to higher-level hospitals. This undermined confidence in the PCN system.

*…sometimes there are no drugs in public facilities that is a challenge*,* and so when you refer clients*,* and then they end up at the pharmacy*,* and there are no drugs*,* then that brings a challenge*,* and so when you refer them again*,* they tell you “you are telling me to go to this place when I go I will just queue and reaching the pharmacy*,* I miss the drugs* **IDI8_Male Community Health Promoter (CHP) County A**

*But one of the biggest challenges that we have in terms of PCN implementation is infrastructure and equipment. Infrastructure because as we expand our scope to provide comprehensive services*,* we need to ensure that we have a matching infrastructure. So*,* I think it’s very critically that we look at that.* **IDI1_Female PHC Coordinator County B**

**PCN health facilities across all the study counties lacked an integrated procurement and supply chain for health commodities**. Key supply chain functions, such as quantification, forecasting, and ordering, were not coordinated and integrated within PCNs. Each health facility in the PCN conducted quantification and ordering independently, with levels 2 and 3 receiving support from the sub-county pharmacist. Furthermore, hub facilities were expected to support spokes by sharing laboratory and radiology services, offering diagnostic tests, and establishing sample referral networks for facilities that lacked these services. However, across the study counties, the sharing of commodities was limited. One county reported sharing laboratory services by transporting samples from spokes without laboratory services to the hubs for analysis.

*No*,* it’s done as a facility. So*,* the facility will just place the orders based on their needs and based on available funds*,* which are actually allocated based on the workload. It’s a problem because we have not yet established the workload of the PCN in our spokes.* **IDI10_Male SCMOH County D**

*…when there are fewer patients or less consumption in the lower facilities*,* we borrow from them. And sometimes*,* when we have more in stock*,* we do help them. We use S11…a tool for whenever you want to give out anything from the pharmacy*,* you must have it signed.* **IDI5_Male Medsup County B**

#### Information systems

**PCN health information systems were partially digitised.** Existing health information systems included the Kenya Health Information System (KHIS) for data on health facility services, the Logistics Management Information System (LMIS) for managing the health commodities supply chain, and the Community Health Information System (CHIS) for community health interventions. There is a partial digitisation of health records, particularly at higher-level facilities (hubs). However, many Level 2 and Level 3 facilities still rely on manual data collection methods.

*And when you find that they are digitised*,* you only find that maybe only other departments*,* and this is probably in some hubs*,* the mini hubs or the hubs whereby we have maybe the pharmacy*,* the CCC*,* yeah there is that partial system that is being used*,* but we don’t have a complete digitised system within the facilities.* **IDI2_Female County Community Health Focal Person County A**

*…routine data coming to the hospital we have a register where every client is attended to and we ensure he is captured in the registers as the permanent source and then we report every month to the reports we give that is in summary and then from there we send to the HRIO to upload to the HIS that is the data we have. But there is no specific data that we have collected or intended to collect because of the PCN not yet.* **IDI10_Male SCMOH County B**

**Community-Based Health Information Systems (CHIS) have been fully digitised**. CHIS was digitised across all the study counties. Community health promoters (CHPs) used e-CHIS to collect and report community data. All CHPs were trained to use e-CHIS. However, while CHPs had access to digital tools, there were difficulties in integrating community data with health facility data, given that most primary healthcare facilities relied on manual systems. Only one study county had started integrating e-CHIS with the information systems used at the health facility level.

*We have*,* like all the Community Health Promoters*,* now it’s digital*,* though now when it gets to the referral*,* as in refer with the system of course*,* now it doesn’t reflect at the facility because the facilities are not digitised.* **IDI2_Female County Community Health Focal Person County A**

*…at the community level*,* they are digitised…household information is collected through the e-CHIS…the CHA and the sub-county focal person helps in ensuring there is quality data being collected from the community before it is keyed in by the sub-county health information record officers into the Kenya Health Information System (KHIS).* **IDI1_Male PHC coordinator County A**

**Health information systems were not integrated across PCN health facilities**. A significant challenge in PCN implementation was the lack of integration and interoperability across the existing national HIS. Although PCNs leverage platforms such as KHIS, eCHIS, and various electronic medical records (EMRs), these systems were often operating in silos, limiting seamless data exchange and referral tracking. Only one of the study counties had a partially functional “smart PCN”, where eCHIS was linked with spoke-level facilities using the Kenya EMR Plus system. However, integration with the hub was constrained by incompatibility with its existing EMR (FanSoft) and political resistance to changing systems. This fragmentation disrupted continuity of care, delayed service delivery, and contributed to inefficiencies in patient management across PCN facilities.

*What I’m grappling with right now is the visibility. Seated here*,* I am not able to see the PCNs because I don’t have an integrated health [information] management system that should actually be in this laptop or in this phone*,* and I can just click and see everything that is happening across the county. I wish I could just be able to open and see what is happening across the county.* **IDI7_Male CECm for Health County D**

*…we are able to see their [CHPs] referrals …. And a system was installed*,* the Kenya EMR plus. …those facilities have Kenya EMR*,* which is working*,* and it was tested that we are able to see the referrals. But now the challenge has come: it is installed on the computer*,* but we are not using it. Because we had a previous EMR that we’re using*,* Fan Soft*,* and there were issues with the management from the county*,* they have not given us the go-ahead to shift from Fan Soft to Kenya EMR*,* so we are not using it. But if we were allowed to use it*,* it has been tested*,* and we are able to do it. It’s interoperable.* **IDI5_Female MDT Team Lead County E**

#### Service delivery

**Strengthened facility-CHU linkages.** Community units were effectively linked to PHC facilities. According to PCN guidelines, PCN facilities should ensure that 100% of the population is covered by CHUs linked to these facilities. In our study, they were indeed linked to CHUs and had designated Community Health Assistants (CHAs) who provided supportive supervision. Additionally, most facilities reported conducting regular community outreach services. The introduction of PCNs has further strengthened these linkages by formalising roles, establishing feedback mechanisms, and providing stipends to CHPs. Moreover, there has been vertical communication from CHPs to policymakers, supporting more responsive health system planning.

*We know who our community health promoters are*,* we interact with them*,* and they also have our contact information. So*,* even at night*,* if a patient needs to come*,* we can be informed*,* and the patient will arrive and know they were being expected. So the community linkage*,* I think*,* with our side it is not so bad*,* we are able to interact*,* we are able even to refer back.* **IDI10_Female Nursing Officer In-charge County A**

*So*,* it [PCN] has greatly improved the linkage between the community units*,* the facility*,* the sub-county team*,* all the way to the county; that linkage has definitely improved. Because when I go*,* for example*,* as a Primary Care Network Coordinator at the county level*,* I know the issues from the Community Health Promoters*,* and these issues are able to be communicated all the way to the county level*,* yeah.* **IDI13_Female PCN Coordinator County C**

**Facility-CHU linkages challenges**. Despite progress, some challenges were faced in integrating CHS within PCNs. First, some spokes lacked active CHPs or had CHPs demotivated by irregular compensation. Second, there were structural issues, such as CHUs being linked directly to hubs instead of spokes or inaccessible spoke locations, which led to the bypassing of lower-level facilities. These gaps undermined the intended referral flow, resulting in overcrowded hubs and inefficient care delivery.

*…the linkage was good*,* but there are some spokes that don’t have any CHPs*,* and I think some have CHPs who are not active …. The linkages are not as strong as they should be … and mainly it was because of demotivation*,* they were not being compensated on time…* **IDI6_Male PHC Coordinator County D**

*… the way the XXX PCN is organised*,* … there are like five community units that are directly attached*,* … there’s no spoke that is near to them. Because they are surrounding here*,* they cannot go to a very far spoke*,* then they come here [hub]… So*,* the referrals were diverted to here.* **IDI5_Female MDT Team Lead County E**

**Care coordination and integration across PCN facilities were inadequate.** For instance, while hub facilities were expected to coordinate with spokes and CHUs to establish a unified catchment population for the PCN across the study counties, each facility independently defined its own catchment area. Furthermore, while counties were expected to establish effective gatekeeping mechanisms to manage patient referrals, no gatekeeping mechanism was in place across all the study counties. Patients could seek PHC services at any facility regardless of the level of care within the PCNs.

*Mostly*,* they [services] are coordinated by the sub-county health management team [SCHMT]. From the level one community health promoters*,* dispensary*,* health centre to the hub that’s the work of the SCHMT.* **IDI1_Male Sub-County Team Lead County E**

*… it’s not really changed much. It’s the same way; we are only insisting that now it has to be in referring across the network; if level two used to refer directly to level four*,* they shouldn’t do that. They should try referring to a level three.* **IDI6_Male PHC Coordinator County D**

**Counties strengthened referral systems within PCNs.** All the study counties strengthened their referral systems, from the community level through the CHPs to the facilities, and across different levels of facilities, using toll numbers and CHP referral desks. Moreover, with support from partners, all the study counties strengthened their emergency referral systems by repairing and outsourcing ambulances.

*Referral mechanism …is an efficient one because there is a toll number to the call centre*,* we refer through the county referral call centre. When there is any critical case*,* we call an ambulance*,* which takes … approximately one hour to arrive here*,* and then the patient is picked up to get services in the referral hospital.* **IDI8_Male Facility Manager Level 3 County D**

*…we have the emergency operation call centre in XXX*,* and the PCNs have ambulances that are designated to them. And those ambulances are not designated to a hospital; they are for the whole PCN.* **IDI8_Male Medsup County A**

**There was inadequate engagement of private healthcare facilities.** There has been insufficient integration of private facilities within PCNs. This gap stemmed from the lack of formalised structures that could facilitate more effective collaboration between the public and private sectors, particularly in areas where private health facilities could relieve pressure on public services. This lack of integration limits the potential reach and effectiveness of the PCN model, which could otherwise benefit from more robust resource sharing and patient load distribution across sectors.

*We’ve not really had very concrete interactions with the private sector yet…it’s good to have them on board because their facilities can serve a huge number of patients within the population.* **IDI6_Male PHC coordinator/SCMOH County D**

*…one of the gaps that has not really been addressed with these PCNs is the involvement of private and faith-based sectors…how they are going to be involved within PCN.* **IDI1_Male PHC coordinator County A**

**Multiple structural and systemic barriers impeded effective PCN service delivery across the study counties.** Key challenges included chronic shortages of human resources, especially in lower-level facilities often run by a single staff member; inadequate infrastructure and basic equipment; frequent stockouts of essential medicines; and poor integration of referral and health information systems. Financial constraints were common, affecting outreach, training, and MDT deployment, as there was no dedicated PCN budget in most counties. Geographic inaccessibility, difficult terrain, and patient cost burden further limited access to care. Political interference and limited stakeholder buy-in compounded these issues. Mitigation efforts included reallocating commodities from hubs to spokes, leveraging CHPs for referrals, providing stipends and refresher trainings, advocating for PHC fund decentralisation, and engaging county leadership in budget planning.

*Like in terms of providing the essential services*,* like a lab*,* I cannot refer a patient from a dispensary*,* take him to a health center. If the dispensary and health center are still offering the same services is there a need for you to refer back to?* **IDI10_Male SCMOH County B**

*I can say the challenges that are there are probably in terms of services*,* take for example a pregnant mother who wanted to do ANC profiling at the nearest facility that will be able to be referred to is a dispensary where they don’t have a lab. So*,* that of course now makes them move to probably elsewhere where they would prefer or where those services are. The other is because of drugs*,* they come for treatment*,* and they don’t get to get the drugs so they would prefer elsewhere where they get all these within the reach. Then I would say another one is the staffing*,* yeah where you have one staff who is constrained so sometimes*,* they prefer elsewhere where they wouldn’t take a lot of time.* **IDI2_Female County Community Health Focal Person County A**

### Facilitators of PCN implementation

**Political goodwill and leadership**. Political support and leadership, both at the national and county levels, were identified as a crucial factor enabling the successful establishment of PCNs. Several actions taken by the current government have strengthened PCN efforts, notably the integration of PHC into the government’s manifesto and the enactment of the PHC Act in 2023. This culminated in the launch of the *Afya Nyumbani* [health in your households] initiative in October 2023, a UHC initiative that is hinged on strengthening facility-based PHC and CHS. This commitment to improving PHC services facilitated the scaling of PCNs across the country.

At the county level, the involvement of governors, county executives, and other political leaders played a pivotal role in initiating PCN implementation. For instance, in one of the study counties, the governor’s personal commitment, shaped by international experiences, directly supported the establishment of PCNs. This political goodwill not only enabled financial allocation but also fostered cross-sectoral collaboration, which is crucial in prioritising resources in environments with limited funding.

*Number one is political support from the Governor*,* right from the word go*,* through the CEC and basically the political class; the Governor was into the idea*,* so it means if he is supporting*,* then it’s going to be implemented.* **IDI1_Male PHC coordinator County A**

*I think the biggest success factor was how invested the political teams were. You had a deadline*,* and you had to report to the President. My friend*,* there is no bigger motivator*,* you have to give feedback to the President*,* and you know like the CS’s jobs hang on the lines for such things.* **IDI2_Female MNH Lead Partner B**

**Non-state partners’ support and collaboration.** The implementation of PCNs has been greatly aided by the collaborations between the government and non-state partners. This collaboration facilitated the mobilising of financial resources, and the technical support required to establish PCNs.

*The stakeholders here are the NGOs that have worked with us*,* the universities*,* medical universities*,* the pharmaceutical companies that have worked with us to try and ensure this is finally achieved*. **IDI8_Male Medsup County A**

## Discussion

This study set out to examine the emergence and early implementation experience of PCNs in Kenya. The introduction of PCNs in Kenya illustrates how the problem, policy, and political streams in Kingdon’s multiple streams framework converge and leverage a policy window to lead to policy emergence [19–21]. Longstanding challenges in PHC service delivery led to the development and piloting of PCNs as a solution in 2020 during the previous government. The PCN reform subsequently gained momentum when it was prioritised by the political class in 2023 as part of the new government’s healthcare reform agenda. This finding aligns with previous observations in Kenya and other settings, where major health systems and UHC reforms have addressed well-defined problems and benefited from being part of a broader political agenda [22–24]. The findings underscore the need for policy entrepreneurs to align specific policy solutions with political interests and to mobilise political support for reforms.

Kenya’s PCNs reflect a growing shift towards networks of care (NOCs) in low- and middle-income countries (LMICs) that link services and providers to improve service integration, referral efficiency, continuity and quality of care [13, 25–34]. Like Ghana’s Networks of Practice, Kenya’s PCNs use a hub-and-spoke structure to connect facilities and support peer learning across the system [35]. Ethiopia, by contrast, places a greater emphasis on community health workers, with weaker connections to facility-based services [36]. What sets Kenya apart is its strong institutionalisation of law through the PHC Act 2023, the inclusion of family physicians in care teams, and growing investment in digital technology for real-time referrals and coordination [8, 14].

Various legislative and policy frameworks enabled the implementation of PCNs in Kenya. These institutional frameworks were critical in legitimising the reform, providing policy and legal backing, and providing guidance for the design and implementation of the reforms. The role of institutional arrangements in policy effectiveness has been recognised [37]. For instance, legal and policy frameworks supported the institutionalisation of healthcare priority-setting reforms globally [38].

While the support of strong non-state actors was an enabler of the PCN reform, the over-reliance on external support poses a sustainability challenge to PCN reforms, especially in a context where counties scarcely allocated domestic resources to implement the reform. External actor reliance on technical and financial support for reforms is prevalent in low- and middle-income countries (LMICs) [39]. This reliance has been shown to crowd out domestic resource allocation, compromising local priorities and the sustainability of reforms [39]. To enhance sustainability, PCNs should be integrated into domestic planning and public financing frameworks.

Implementation effectiveness is a critical determinant of the success of health policy reforms [40, 41]. Often, well-designed policies fail due to suboptimal policy implementation. Effective implementation, among others, hinges on policy capacity, which encompasses political, technical, and operational dimensions [42–45]. While this study found sufficient political capacity to rally support for PCN reforms and technical capacity for policy design, gaps remain in operational capacity. The study found that implementation fidelity varied across study counties. While study counties made progress in introducing PCNs by implementing most of the prescribed implementation steps, several critical gaps remained. These included limited sensitisation of lower-level health teams, incomplete formation of structures such as MDTs and PCN committees, inadequate assessments of client and resource needs, and digitisation. These shortcomings likely stem from rapid scale-up without sufficient learning from the pilot phase or adequate county-level capacity building. The accelerated rollout, while politically expedient, may have compromised depth and quality, resulting in uneven readiness across counties and potential inefficiencies in service delivery. This highlights the need for phased implementation grounded in continuous learning and capacity support. Moreover, this was also emphasised by the observation that PCN implementation in Kenya has mainly relied on the technical and financial support of non-state partners. As Kenya scales up PCNs, strategic investment is needed to strengthen operational capacity at the county level to ensure consistent implementation fidelity and reform effectiveness.

Another observation is the inadequate underlying PHC health system capacity that characterises PCNs in Kenya. PCNs are expected to improve the delivery of PHC services by targeting healthcare organisations to enhance performance [13]. For PCNs to be effective, the underlying functionality of health systems should be assured. Put another way, the reorganisation of service delivery is unlikely to achieve much if health facilities do not have medicines, health workers, basic equipment, and robust information systems. PCN implementation in Kenya reveals that inadequate investments in foundational aspects of health system capacity, such as health workers, medicines, and infrastructure, can compromise the success of well-intentioned health system reforms by reducing their effectiveness. It highlights the need for whole-system reforms, where a primary reform is accompanied by supportive and complementary interventions. Failure to do this can undermine the intended effect of reforms. For instance, the poor capacity of lower-level PHC facilities led patients to bypass them for high-level facilities, undermining the intention of PCNs to promote efficiency by strengthening the referral mechanism and gatekeeping. Poor underlying health system capacity can also lead to unintended policy effects. For example, the establishment of MDTs, which were intended to provide outreach services within PCNs, without strengthening the staffing complement of hub facilities, resulted in disruptions to health services at hub facilities. The importance of using a whole-system approach to health system reforms has been demonstrated in other settings [46, 47].

Lastly, the findings show that PCNs in Kenya had limited functional integration. The anticipated impacts of networks of care, which include improvements in quality of care, equitable access to healthcare, and efficiency of service delivery, are hinged on collaboration and interconnectedness among the facilities within the networks [13, 48]. While the networks of care literature emphasise coordination of care delivery [13, 48, 49], our findings show that the coordination of care should be underpinned by the integration of key health facility functions. For instance, it is difficult to coordinate care and ensure continuity of care if information systems across networks of care are fragmented rather than connected. We found that key functional elements of health facilities in the PCNs were not integrated. These include financing and planning, human resource management, commodities and supply chain management, information systems, and care delivery. The fact that these functions were not integrated and coordinated meant that PCNs were networked *de jure* but operated with little or no coordination *de facto*. This highlights the need for policy revisions that facilitate the integration of core health system functions such as joint annual planning across PCNs, pooled financing at the network level, shared deployment of HRH, integrated procurement and supply chain systems for essential commodities, and interoperable digital health information platforms.

This study has several strengths that enhance the validity, depth, and policy relevance of its findings. First, the inclusion of diverse stakeholders across national, county, facility, and community levels provided rich multilevel perspectives on PCN implementation. Second, the study was guided by a robust conceptual framework that enabled a structured analysis of foundational capacity, functional integration, and implementation fidelity, key dimensions influencing reform effectiveness. Third, the purposive selection of counties representing geographic, demographic, and implementation diversity increased the transferability of insights across Kenya’s health system. Finally, the study was conducted at a pivotal moment during the early phase of national PCN rollout, making its findings timely and strategically aligned with ongoing PHC reforms.

This study had some limitations. First, we did not assess critical relational factors like collaboration, teamwork, trust, and communication. These are crucial for delivering high-quality PHC. We focused on the structural aspects of PCN implementation. These are key to preparing the health system to provide PHC services. Future research should explore the relational factors that impact the effectiveness of PCN implementation. Second, the study was conducted early in the implementation of the PCN reforms. The implementation outlook would likely be different after several more years of implementation. Finally, while we assessed the perceived impact of PCNs, many respondents noted it was too early to draw firm conclusions. Reported effects were mixed and, in some cases, contradicted preliminary quantitative findings (to be reported separately), which showed no significant impact of PCNs. These limitations underscore the need for longitudinal research to gain a deeper understanding of how PCNs evolve and impact health system performance over time.

To strengthen PCN implementation in Kenya and similar settings, a phased and prioritised approach is essential. As a top priority, national and county governments should allocate sufficient resources to support PCN implementation by integrating PCNs into county health budgets. This includes conducting comprehensive resource needs assessments to inform planning and reduce overreliance on partner funding, which threatens sustainability. Next, counties should ensure the full operationalisation of PCN design elements, such as comprehensive stakeholder sensitisation, the establishment of governance structures including PCN committees and MDTs, and rapid digitisation to enhance coordination and accountability. Concurrently, critical service delivery gaps must be addressed by dedicating budgets to recruit and retain health workers, procure essential drugs and supplies, upgrade infrastructure such as laboratories and referral transport, and invest in digital tools for HIS integration. Finally, functional integration mechanisms must be developed to operationalise PCNs as coherent units within health systems. This includes adapting public finance management and reimbursement frameworks to support networked service delivery, integrating commodity and information systems for shared resource use, and enabling staff sharing across facilities to support coordinated and continuous care.

## Conclusion

The implementation of PCN reform is a key strategy in Kenya’s PHC-focused UHC reforms. The reform has received substantial political and stakeholder support and is facilitated by a robust legislative and policy framework. Experiences from other settings show that PCNs as a NOC intervention have the potential to improve PHC service delivery [13, 48, 49]. Our study shows that for Kenya to realise these potential gains, attention should be paid to strengthening policy design to ensure functional integration within PCNs, enhancing implementation fidelity through strengthening implementation effectiveness, and investing in foundational health system capacity.

## Supplementary Information

Below is the link to the electronic supplementary material.

Supplementary Material 1

## Data Availability

The dataset from this study will not be shared publicly for ethical reasons, including maintaining participants’ confidentiality and anonymity. The data will be available through a formal request to the corresponding author on reasonable request.

## Abbreviations

AWP: Annual Work Plan
CECM: County Executive Committee Member
CHC: Community Health Committee
CHIS: Community-based health information system
CHMT: County health management team
CHP: Community Health Promoter
CHS: Community Health Services
CHU: Community Health unit
CHW: Community Health Worker
eCHIS: Electronic Community-based Health Information System
FBO: Faith Based Organization
IDI: In Depth Interviews
KHIS: Kenya Health Information System
LMICs: Low-and-Middle Income Countries
LMIS: Logistics Management Information System
M&E: Monitoring and Evaluation
MDT: Multi-Disciplinary Team
MoH: Ministry of Health
NACOSTI: National Commission for Science, Technology, and Innovation
PCNs: Primary Health Care Networks
PFM: Public Finance Management
PHC: Primary Health Care
SCHMT: Sub-County Health Management Team
SCMOH: Sub-County Medical Officer of Health
SERU: Scientific and Ethics Review Unit
TWG: Technical Working Group
UHC: Universal Health Coverage
USD: United States Dollar
CDH: County Director of Health
EMR: Electronic Medical Records
SHA: Social Health Agency
SHIF: Social Health Insurance Fund
NHIF: National Health Insurance Fund
PATH: Program for Appropriate Technology in Health
UNICEF: United Nations International Children’s Emergency Fund
THS: Transforming Health Systems
USAID: United States Agency for International Development
NGO: Non-Governmental Organisation
Medsup: Medical Superintendent
