# Supplementary material for: Examining the emergence and implementation experience of Primary Health Care Networks (PCNs) in Kenya: a qualitative process evaluation
Source: BMC Health Serv Res. 2025 Nov 18;25:1479. doi: 10.1186/s12913-025-13652-2 (PMC12625690; doi:10.1186/s12913-025-13652-2)
Supplement: Supplementary file 1 — Supplementary Material 1 [file 12913_2025_13652_MOESM1_ESM.pdf]

**In-depth Interview Guide**

Study: **Evaluating the implementation experience of Primary Health Care Networks in Kenya**

| | |
|---|---|
| **KII ID:** | |
| **Date (DD/MM/YYYY)** | |
| **County** | |
| **Gender** | |
| **Job Title** | |
| **Name of Organization** | |

Thank you for consenting to participate in this study via the interview that will be audio recorded. In this interview, we value your honesty. There is no right or wrong answer because our interview concerns your experience with PCN implementation. For now, let us begin our interview.

| **Domain** | **Interview questions** |
|---|---|
| **Introduction** | i. Please describe your current role in this country/county/facility. How long have you worked in this role?<br>ii. What do you consider as primary health care within your county/facility?<br>iii. Have you heard of Primary Care Networks? If yes, please tell me how you understand it. |
| **The emergence of PCN reforms** | **I would like to understand how PCNs were established in this country/county/facility?**<br>*Probe:*<br>• *When did the PCN implementation begin? How?*<br>✓ *Why was it necessary for the country/county/facility to establish PCNs?*<br>• *Who established PCNs?*<br>✓ *Probe for organizations, partners, other key stakeholders*<br>✓ *What roles did they play? What were their key priorities/interests?*<br>▪ *What's is the role of the county? National MOH?*<br>▪ *Do they have the same priorities when making decisions regarding PCN implementation?*<br>*-If not, how do they differ? Why?*<br>*-How do different priorities get balanced?*<br>• Who is supporting PCN implementation in this county?<br>✓ Probe for donors/technical support<br>✓ What form of support are they offering – TA? Financial? |

| Domain | Interview questions |
| --- | --- |
| | ✓ What are the coordination mechanisms of partners/stakeholders? Private and other sectors?<br>• Were you engaged in the design and implementation of PCNs? How? *What roles did you play?* |
| **PCN implementation process** | **What are the design features of PCNs in this county?** *(what/how/why?)*<br>✓ ***Governance and accountability***<br>▪ *What is the governance arrangement for PCNs?*<br>▪ *What committees exist (e.g. a functional PCN Committee)*, How were they established; *what are their roles? Are these roles formally defined? Why or why not? Do they meet and how frequently? When was the last time they met;* who are the members and *what is the gender representation?*<br>▪ *Is there a leadership/governance/oversight committee/team for PCNs?*<br>▪ *What are the reporting mechanisms? monitoring and supervision mechanisms?*<br>▪ *Are there PCN performance indicators or targets to assess the impact?*<br>▪ *Are the governance mechanisms effective? What challenges do they face?*<br>▪ *Are spokes accountable to hubs?*<br>▪ *Have those in leadership positions received any leadership and managerial training?*<br>✓ ***Financing,***<br>▪ *How are PCNs financed? What are their sources of funding?*<br>▪ *How are coordination, supervision, monitoring, and governing activities of PCNs financed? Any challenges here?*<br>▪ *Do PCNs pool their funding? Do they have separate or one bank account?*<br>▪ *Do facilities in PCNs prepare separate or one annual work plan? Is there a consolidated PCN plan?*<br>▪ *Do facilities prepare separate or one budget? Is there a consolidated PCN budget?*<br>▪ *Do PCNs make joint claims to NHIF or separate claims?*<br>▪ *Are PCN facilities reimbursed individually by NHIF or are they jointly reimbursed?*<br>▪ *Is there a budget allocated for the PCN implementation?*<br>• *What are the PCN establishment costs?*<br>• *What are the PCN operational costs?*<br>▪ *What is the process for developing and approving the budget for the PCN reform?*<br>▪ *Do you have a costed annual PCN work plan? What does it entail*<br>*Overall, what has been the impact of PCNs on;*<br>• *On the planning and budgeting process?*<br>• *Absorption and utilization of funds?*<br>✓ ***Health workforce,*** |

| Domain | Interview questions |
| --- | --- |
| | ▪ *What is the formal mechanism (in policy or guidelines) for coordinating human resources across PCN facilities (hub and spokes)*<br>▪ *How are HRH organised within your PCNs?*<br>▪ *What are the challenges with this mechanism?*<br>▪ *Do hubs and spokes share staff? For instance, does a clinician in the hub also see patients in a spoke? Do staff rotate across facilities within the hub and spoke?*<br>▪ *Are you aware of MDT? How do you understand it?*<br>• *How was it established? Where is it located? Who are the members? Who is the team lead? How many members are female?*<br>• *What are their roles? Are these roles formally defined? Why or why not?*<br>• *What are their competences?*<br>• *What is the skill mix?*<br>• *what services do they provide and where? (hub/spoke/community/rest of the HS)*<br>• *How functional/effective are the MDTs? What challenges do they face?*<br><br>*Overall, what impact has the PCNs had on availability, capacity, and motivation of staff? (distribution/supervision?)*<br>✓ ***Health commodities,***<br>▪ *What is the formal mechanism (in policy or guidelines) for coordinating commodity supply chain across hubs and spokes?*<br>▪ *What are the challenges with this mechanism?*<br>▪ *How do PCN facilities order commodities, do they do it separately or jointly?*<br>▪ *How do PCN facilities do quantification and forecasting? Do they do it separately or jointly?*<br>▪ *Do PCN facilities have visibility over stocks in each health facility?*<br>▪ *Is there a formal mechanism (in policy or guidelines) for sharing commodities – distributing and re-distributing- across PCN facilities? how does it work, what are the challenges*<br>▪ *Are their informal mechanisms?*<br>• ***Are PCN facilities well equipped to deliver services? Please explain***<br>o *Are there stockouts? Can you provide specific examples of commodities that are frequently in short supply in health facilities? Why? How do you address stockouts?*<br><br>*Overall, what impact has the PCNs had on the supply and availability of commodities in health facilities?* |

| Domain | Interview questions |
|---|---|
| | ✓ ***Service delivery,***<br>▪ *What is the service delivery design within a PCN?*<br>▪ *Understanding what is a hub? What is a sub-hub? What is a spoke?*<br>▪ *What services are offered at the Hub? At the spoke?*<br>▪ *How is service delivery coordinated across PCNs?*<br>▪ *Are there formal mechanisms (in policy or guidelines) to coordinate services?*<br>▪ *What mechanisms, systems and processes facilitate continuity of care*<br>▪ *What gate keeping mechanisms exist and how effective are they?*<br>• *Do you have a gatekeeping policy? What are the main goals of the policy?*<br>▪ *How effective is the referral mechanism?*<br>• *Do you have a referral policy? What are the main goals of the policy?*<br>• *Is it regularly reviewed?*<br>o *Which stakeholders are part of the referral policy technical review team? What are their roles?*<br>▪ *Could you please describe how the PCN facilities are integrating the delivery of care? What is connection with the rest of Health System?*<br>✓ ***Information and research?***<br>▪ *Do you collect/report any information in the PCN? What kind of information is collected?*<br>▪ *How does information flow between hubs and spokes?*<br>▪ *Do PCN facilities have an integrated health information system? Can someone in a hub see what patients and services are offered in a spoke?*<br>▪ *Is there data linkage between hubs and spokes to facilitate continuity of care? how does it work?*<br>▪ *Who has access to the skills, devices and technology that transmits and processes health information?*<br>▪ *Is this information used? (at the hub/at the spoke/county/national) How?*<br>▪ *Are the PCN digitized? How?*<br>***What is the size of the PCN? What is the model?***<br>• *Who are included in a PCN? (No of HFs/type/ownership/level)*<br>• *What is the catchment population?*<br><br>**Kindly describe how PCNs are being implemented in the country/county/facility?**<br>✓ What steps were used to establish PCNs? (activities involved in establishing PCNs)<br>Probe whether they did the following? |

| Domain | Interview questions |
|---|---|
| | 1. *Stakeholders' sensitization; partners, private, FBOs, CECs, CHMT & SCHMT*<br>2. *Establishment of governance and coordination structures including MDTs*<br>3. *Conducting a baseline assessment of community health needs, PCN facilities needs, resources and partners*<br>4. *County presentation of its PHC implementation*<br>5. *Dissemination of baseline assessment*<br>6. *Mapping of the hubs to serve a population in a defined* geographical area<br>7. *Mapping of spokes and CHUs, and linking them to the hubs*<br>8. *Mapping and registration of households, taking into account the health profiles of each household member (health status profiling)*<br>9. *Identification of the financial requirements to establish and management of a PCN*<br>10. *Setting up of M$E systems for monitoring the PCNs*<br>11. *Digitization of PCNs-eCHIS, telemedicine, record sharing- e-payments*<br>12. *Gazettement of PCNs*<br>• What laws and regulations guide the establishment and implementation of PCNs in your county? Please explain how?<br>• Were implementation guidelines put in place for the effective implementation of the PCN reforms? If not, why not? If yes; *(PCN/County specific/informal processes)*?<br>*Probe:*<br>✓ *Where were these guidelines adapted from? how?*<br>• Did the implementation of the PCNs deviate from what is described in the national guideline? How? Why?<br>• Were there any parts of the PCN implementation plan/policies etc that were not implemented or partially implemented? Which ones? Why?<br>• How are the policies and guidelines communicated to the different stakeholders involved in PCN implementation?<br>• Is there a training or orientation process to ensure understanding and compliance? (capacity building)<br>✓ How do you ensure that the policies and guidelines are consistently followed? |
| **Facilitators and barriers** | ✓ **What factors have facilitated the successful implementation of PCNs in the country/county?**<br>✓ **What challenges/constraints/barriers have you encountered while implementing PCNs? How were these challenges addressed?** |

**Thank you for your time and participation in this study!**
